# Systematic review on women's values and preferences concerning breast cancer screening and diagnostic services

**DOI:** 10.1002/pon.5041

**Published:** 2019-03-24

**Authors:** Alexander G. Mathioudakis, Minna Salakari, Liisa Pylkkanen, Zuleika Saz‐Parkinson, Anke Bramesfeld, Silvia Deandrea, Donata Lerda, Luciana Neamtiu, Hector Pardo‐Hernandez, Ivan Solà, Pablo Alonso‐Coello

**Affiliations:** ^1^ Biomedical Research Institute (IIB Sant Pau) Iberoamerican Cochrane Centre Barcelona Spain; ^2^ Division of Infection, Immunity and Respiratory Medicine, Faculty of Biology, Medicine and Health University of Manchester Manchester UK; ^3^ Department of Public Health, Faculty of Medicine University of Turku Turku Finland; ^4^ Joint Research Centre European Commission Ispra Italy; ^5^ Clinico‐Pharmacological Unit Finnish Medicines Agency Fimea Turku Finland; ^6^ Institute for Epidemiology Social Medicine and Health System Research Hanover Medical School Hannover Germany; ^7^ Health Protection Agency Metropolitan city of Milan Italy; ^8^ CIBER de Epidemiología y Salud Pública (CIBERESP) Barcelona Spain

**Keywords:** breast cancer, cancer, diagnostic services, oncology, patient‐centred care, patient preference, practice guideline, screening

## Abstract

**Background:**

There is still lack of consensus on the benefit‐harm balance of breast cancer screening. In this scenario, women's values and preferences are crucial for developing health‐related recommendations. In the context of the European Commission Initiative on Breast Cancer, we conducted a systematic review to inform *the European Breast Guidelines*.

**Methods:**

We searched Medline and included primary studies assessing women's values and preferences regarding breast cancer screening and diagnosis decision making. We used a thematic approach to synthesise relevant data. The quality of evidence was determined with GRADE, including GRADE CERQual for qualitative research.

**Results:**

We included 22 individual studies. Women were willing to accept the psychological and physical burden of breast cancer screening and a significant risk of overdiagnosis and false‐positive mammography findings, in return for the benefit of earlier diagnosis. The anxiety engendered by the delay in getting results of diagnostic tests was highlighted as a significant burden, emphasising the need for rapid and efficient screening services, and clear and efficient communication. The confidence in the findings was low to moderate for screening and moderate for diagnosis, predominantly because of methodological limitations, lack of adequate understanding of the outcomes by participants, and indirectness.

**Conclusions:**

Women value more the possibility of an earlier diagnosis over the risks of a false‐positive result or overdiagnosis. Concerns remain that women may not understand the concept of overdiagnosis. Women highly value time efficient screening processes and rapid result delivery and will accept some discomfort for the peace of mind screening may provide.

## 1 INTRODUCTION

Breast cancer is the most common cancer in women and one of the leading causes of all cancer deaths both in Europe and worldwide.^1^ Breast cancer screening with mammography, the only population‐based method for the early detection of breast cancer currently used, has been shown to reduce breast cancer mortality in women aged 50 to 74 years^2^ and is widely implemented in most European countries.^3^ However, mammography screening is also associated with potential important undesirable effects, including overdiagnosis, and hence overtreatment, and false‐positive mammography results.^4^ False‐positive mammography findings may cause psychological distress.^5^ The balance between benefits and harms of screening becomes less favourable after 74 years of age and at 90; harms are considered to outweigh benefits, largely as a consequence of overdiagnosis.^6^ There is still a lack of consensus on the benefit‐harm balance of breast cancer screening thus underlining the need for women to receive balanced and adequate information in order to make informed decisions concerning their participation in screening programmes.

The European Commission Initiative on Breast Cancer (ECIBC) (http://ecibc.jrc.ec.europa.eu/) uses the GRADE approach when formulating recommendations for breast cancer screening and diagnosis. This includes the use of Evidence to Decision (EtD) frameworks when moving from evidence to recommendations.^7^ The EtD frameworks provide an explicit and transparent system for decision making that can help ensure all important criteria, informed by the best available research evidence, needed to make a decision are considered. One of these criteria is how those affected by a recommendation value the main desirable and undesirable outcomes of the interventions considered. In the case of recommendations on breast cancer screening, this means considering women's values and preferences regarding potential consequences of participating in screening.

Women's values and preferences refer to the relative weight those affected by a recommendation place on the different outcomes, such as the potential benefits, harms, costs, limitations, and inconveniences of the available interventions or management options.^8^ Inclusion of women's values in the screening decision making process has been proposed for decades now,^9^ but its implementation is still suboptimal. GRADE's EtD frameworks provide guidance on how to incorporate women's values and preferences while drafting clinical recommendations. This systematic review was thus conducted to inform ECIBC's clinical recommendations' development process.

## 2 METHODS

### 2.1 Design

A systematic literature review, following standard Cochrane Collaboration methodology,^10^ was performed to address the following question: What are the values and preferences of women regarding decision making on breast cancer screening and diagnosis. The review protocol is registered in PROSPERO (https://www.crd.york.ac.uk/PROSPERO/display_record.php?RecordID=41487).

### 2.2 Search strategy and selection criteria

Medline (assessed through Ovid) was searched using terms regarding breast neoplasm/cancer; screening; diagnosis; different screening and diagnosis outcomes; values, and preferences (complete search strategy in Appendix 1). As a source for individual studies, systematic reviews with no time restrictions were searched. For primary studies, publications from 2006 until the end of June 2018 were included. Only studies in English were included.

Only studies examining women's preferences for breast cancer screening versus no screening or about the potentially available breast cancer diagnostic alternatives, studies evaluating how women value breast cancer screening and diagnosis outcomes, and those examining the choices women facing a breast cancer screening or diagnostic decision make, when informed about the expected desirable and undesirable outcomes, were included. We excluded studies restricted to women's knowledge, views, behaviours, perceptions, attitudes, and expectations regarding breast cancer screening and diagnosis. We also excluded those conducted in countries outside the Organisation for Economic Co‐operation and Development (OECD) or those focusing exclusively on minorities from geographic regions outside Europe.

### 2.3 Screening and data collection

One reviewer screened the search results based on title and abstract. Two reviewers independently confirmed eligibility of relevant articles based on the full text, and disagreement between researchers was solved by the third reviewer. One reviewer extracted the main characteristics and main findings of the included studies in a tabular format. Another reviewer checked the extracted data for accuracy. The synthesis of the results is described narratively and is based on the identification and grouping of themes reported in the included studies.

Risk of bias assessment was carried out using the domains suggested in the GRADE approach for quantitative studies^11^ and the Critical Appraisal Skills Programme (CASP) checklist^12^ for qualitative studies. The confidence (quality or certainty) of the evidence was rated from high to very low considering the standard GRADE domains for quantitative data.^10^ For qualitative studies, the CERQual (Confidence in the Evidence from Reviews of Qualitative Research) approach was used.^13^ The results of the systematic review were reported according to the PRISMA (preferred reporting items for systematic reviews and meta‐analyses) statement.^14^

## 3 RESULTS

**Figure 1:**
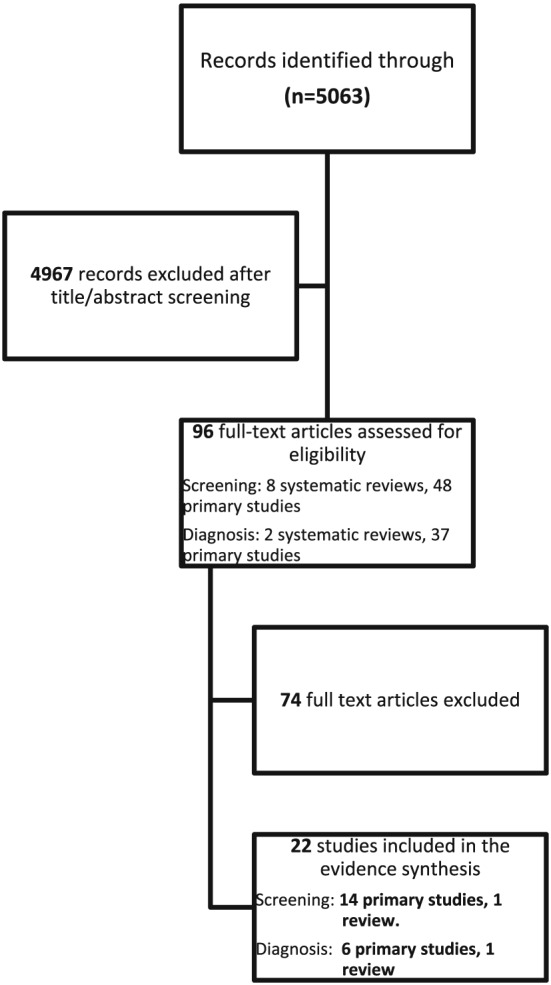
Preferred reporting items for systematic reviews and meta‐analyses flowchart for inclusion and exclusion of the studies

The search yielded 5063 unique references, of which 96 were deemed potentially eligible for inclusion, based on initial screening of titles and abstracts. After full text appraisal, 22 individual studies (15 on screening and seven on diagnosis) involving 12 174 women were included. The PRISMA flowchart is presented in Figure 1. A tabular summary of the findings and rating of certainty of evidence is presented in Table 1. Evidence profiles including main findings and certainty of evidence, for both screening and diagnosis, are included in Appendix 2.

**Table 1:** Tabulated summary of findings and rating of the confidence in the evidence about screening

Review Finding	Confidence in the Evidence	Explanation	Studies Contributing to the Review Finding
*False positives* Women significantly place a low value on the psychosocial and physical effects of false‐positive results. However, women consider false‐positive results an acceptable consequence of mammographic breast cancer screening.	Moderate confidence	There are significant concerns regarding women's lack of understanding about breast cancer screening, especially the undesirable effects. In addition, the adequacy of the information provided to the breast cancer participants, which would help them take an informed decision, seems to be inadequate.	Bolejko,^20^ Bolejko,^21^ Ganott,^23^ Brodersen,^22^ Thompson,^24^ Tosteson,^25^ Vass^26^
*Overdiagnosis* Women significantly place a low value on the psychosocial and physical effects of overdiagnosis. However, women generally seem to consider these undesirable effects acceptable given their knowledge about the potential desirable consequences of breast cancer screening.	Low confidence	There are significant concerns regarding women's lack of understanding about breast cancer screening, especially the undesirable effects. For instance, Van den Bruel et al reported that 10% to 14% of the participants accepted overdetection in the overall population, implying that they did not comprehend the aims of screening and the concept of overdiagnosis. In addition, the information provided to breast cancer participants, which would help them take an informed decision, seems to be inadequate. Also, indirectness is a limitation of some of the included studies, which assessed adult women of any age, rather than women of screening age.	Baena‐Cañada,^15^ Hersch,^19^ Van den Bruel^16^ Waller,^17^ Waller^18^

### 3.1 Screening

We identified the following main themes that contribute to how women value the main outcomes of screening: risk of overdiagnosis and false‐positive screening results, burden of breast cancer screening, and challenges elderly women face when making a decision to participate in screening programmes.

#### 3.1.1 Overdiagnosis

Five studies, four conducted in Europe^15^, ^16^, ^17^, ^18^ and one in Australia,^19^ evaluated women's knowledge and acceptability of the risk of overdiagnosis. Results revealed limited awareness of the risk of overdiagnosis among women. Only 29% and 53% of participants in two population‐based surveys, conducted in the United Kingdom, were aware of the concept of overdiagnosis.^16^, ^18^ In a study from Spain, only 10% of women had adequate knowledge about the implications of being overdiagnosed.^15^

Four studies^15^, ^17^, ^18^, ^19^ assessed the impact exposure to information concerning overdiagnosis has on women of screening age. Information about overdiagnosis and its implications triggered different immediate reactions among participants. These included surprise and concern regarding the undesirable psychological and physical consequences, as well as defensive reactions and mistrust of the investigators' motives. On the one hand, women considered that it would be appropriate and fair to provide adequate information regarding overdiagnosis to women invited for screening but, on the other, they were concerned this information may cause confusion and deter women from participating.

Two studies evaluated the impact information concerning overdiagnosis had on women's intention to participate in screening.^17^, ^18^ Ninety percent of participants answered that they would probably or definitely attend screening in the future. Only 7%, especially women below the recommended screening age, actually showed a decrease in screening intention.^18^

Two studies evaluated the rate of overdiagnosis that women were willing to accept.^16^, ^19^ On the one hand, a survey showed that they were willing to accept 15% and 31% overdiagnosis for an expected benefit of 10% and 50% reduction in cancer specific mortality, respectively.^16^ On the other hand, a focus group study, showed that a rate of 1% to 10% of overdiagnosis was perceived as completely acceptable; 30% was perceived as still acceptable by most women, but 50% was considered to be extremely high.^19^

The willingness to accept overdiagnosis was related to socio‐demographic factors: those with a higher educational status accepted significantly higher levels of overdiagnosis than those with a lower educational status. Furthermore, women over 50 accepted significantly less overdiagnosis than younger women.^16^ However, in another study, younger participants interpreted overdiagnosis as a distinctly negative factor, discouraging them from participating in screening.^19^

The confidence in the evidence regarding overdiagnosis was considered low. There were significant concerns regarding some studies about whether women were adequately informed in order to fully understand the extent of the risks and benefits associated with breast cancer screening, and specifically the implications of overdiagnosis. This can be seen, for example, in a cross‐sectional study evaluating 510 British females, 15% of the participants declared that they were prepared to accept overdiagnosis in the complete population, strongly suggesting that they may have not comprehended the aims of screening and the concept of overdiagnosis.^16^ Indirectness was an additional limitation in some of the studies, as some studies included adult women of any age, rather than women at screening age.

#### 3.1.2 False‐positive screening results

The burden and acceptability of false‐positive mammography screening results was evaluated in seven studies.^20^, ^21^, ^22^, ^23^, ^24^, ^25^, ^26^ A European cross‐sectional study involving 1018 women from the general population used an online discrete choice experiment survey to elicit patients' preferences regarding false‐positive results.^26^ Respondents highly valued the possibility of early diagnosis and were prepared to accept unnecessary follow‐up appointments as a result of a false‐positive screening result. In fact, over 60% of participants were prepared to accept a 20% false‐positive rate for a 3% probability of detecting cancer.

Two longitudinal European studies^21^, ^22^ included a pooled population of 671 patients with false‐positive screening results, 174 patients diagnosed with breast cancer and 1363 matched women with negative results. Here, a false‐positive mammography screening result was associated with consistently greater negative psychosocial consequences compared with a negative result, even 3 years after final diagnosis. However, a study conducted in the United States^25^ found only a transient increase in personal anxiety after false‐positive results, which did not persist at one year after final negative diagnosis was made.

Four studies assessed women's attitudes and beliefs on the effects of false‐positive mammograms towards future screening behaviour. Ganott and colleagues^23^ reported that, prior to mammography examination, 97% of women believed a false‐positive result would not deter them from screening. Tosteson et al^25^ reported that among women with a previous false‐positive mammography finding, the future screening intention was significantly increased compared with those with a negative mammogram. These findings were confirmed by two qualitative studies including women with false‐positive mammography results.^20^, ^24^ A significant proportion of women would accept the inconvenience and anxiety associated with a higher recall rate if this implied the possibility to detect breast cancer earlier.^23^

The confidence in the evidence from the cross‐sectional studies regarding false‐positive findings was moderate because of methodological limitations (significant concerns regarding inadequacy of information provided to participants that led to poor understanding of benefits and risks of breast cancer screening). The confidence in the evidence from qualitative studies was low, as there were similar methodological limitations, but also these studies mostly evaluated preferences of women who had already received a false‐positive result and their preferences may not be representative of the general population of women at screening age. Based on all available evidence, the confidence in the evidence was moderate.

#### 3.1.3 Burden

A metasynthesis^27^ including 21 qualitative studies, assessed barriers for breast cancer screening from the women's perspective. The authors reported several aspects of breast cancer screening that may be burdensome for women including: logistical implications, such as investing time and money to reach the screening site, psychological distress associated with the screening process itself, derived from fear of a positive result, embarrassment, and from not receiving services in line with their cultural and religious beliefs. The confidence in the evidence was moderate being limited by the insufficiency of data and methodological limitations.

#### 3.1.4 Screening decisions among elderly women

Two studies in the United States assessed factors that influence the decision of elderly women (aged 80 and over) to participate in screening programmes.^28^, ^29^ A qualitative study highlighted a more pronounced variability in elderly women's preferences.^28^ Factors influencing more their decision to be screened included women's perceived individual risk of breast cancer, physician's advice, previous screening habits and experiences with mammography, as well as social and family influences. The most important reasons for declining screening were the decision not to undergo a possible operation given their age, and the discomfort associated with an additional clinical visit.^28^ In a cross‐sectional study, women aged 80 and older who decided not to undergo breast cancer screening, ranked their age and doctor's counselling as the factors mostly influencing their decision.^29^

### 3.2 Diagnosis

#### 3.2.1 Anxiety

One of the main themes concerning diagnostic procedures in breast cancer is the avoidable anxiety, mostly because of inadequacy of the information regarding procedures and the delay in receiving test results. This theme was reported in four cross‐sectional studies.^30^, ^31^, ^32^, ^33^ one qualitative study,^34^ and one systematic review^35^.

Women highly valued receiving diagnostic results in a timely manner. Twelve percent of women, who underwent image‐guided breast biopsies in the United States, were not even satisfied with a 1‐day waiting time for their results. However, 90% of them found receiving the test results over the phone to be acceptable if that accelerated the process.^30^ A cross‐sectional study including women who had previously undergone sentinel node biopsy with intraoperative diagnosis found similar results; 95% of participants would choose to undergo the procedure again in the future, in order to have the results earlier.^31^ Another cross‐sectional study showed that better communication with the radiologist performing the biopsies was associated with lower post‐biopsy anxiety.^32^

A systematic review showed that the needs for supportive care concerning diagnosis touch upon many domains, which cluster around psychological and information needs. These needs are influenced by individual clinical, demographic, emotional, psychological, or psychosocial characteristics of subjects.^35^ Finally, one study, including only women aged 60 and over, provided information on the benefits of a decision aid.^33^ The authors did not find any significant differences in decisional support needs based on age at diagnosis, education level, ethnicity, or presence of comorbidities. Approximately 90% of women indicated they had received a high level of support during their cancer diagnosis. However, the desire for additional educational resources such as worksheets, consultation summaries, or workbooks to assist treatment decisions was highlighted. The overall confidence in these findings concerning anxiety is moderate because of inadequacy of data.

#### 3.2.2 Inconvenience

As part of a trial in Australia, a cross‐sectional study with 49 women assessed their experience with contrast‐enhanced spectral mammography (CESM) compared with contrast‐enhanced magnetic resonance imaging (MRI) (CEMRI) during preoperative breast cancer staging.^36^ Significantly higher overall preference towards CESM was shown, with faster procedure time, greater comfort, and lower noise level cited as the commonest reasons. Participants also reported significantly lower rates of anxiety during CESM compared with CEMRI. The overall confidence in these findings is moderate because of inadequacy of data.

## 4 DISCUSSION AND CONCLUSIONS

### 4.1 Main findings

Our review shows that women place a low value on the psychosocial and physical effects of overdiagnosis and false‐positive mammography screening results, as well on the inconvenience and burden associated with it. Women generally consider these undesirable effects acceptable, recognising the potential benefits of breast cancer screening. However, the confidence in the evidence supporting these findings is low to moderate because of methodological limitations. Regarding diagnosis, women highly appreciate avoiding anxiety caused by delays in the receipt of results or suboptimal communication with health care professionals. They also appear to value faster procedures over the inconvenience associated with them.

### 4.2 Our results in the context of previous results

#### 4.2.1 Overdiagnosis

The level of overdiagnosis that women were willing to accept was relatively high, up to 30%.^16^, ^19^ The most commonly reported estimates of overdiagnosis from screening programmes are around 10% and vary widely.^4^ Thus, the level of overdiagnosis women were willing to accept was on the high end of the estimated average figures. The high rates of overdiagnosis women were willing to accept could put into question whether the concept was really understood by study participants. According to our review, women's knowledge and understanding concerning overdiagnosis were variable, and in general limited, with only about 30% to 50% of women being aware of the concept and only 10% having adequate knowledge about its implications. Results from a recently published study from the United Kingdom revealed that almost one‐third of participants reported having previously encountered the term overdiagnosis, but responses often indicated they had very limited knowledge about its implications.^37^

Women appear to overestimate the benefits of mammography screening. Up to 70% of women overestimated the possibility of having breast cancer detected during screening.^23^ The fear of getting breast cancer may also lead women to be willing to accept a higher level of overdiagnosis. Population‐based studies have consistently shown that between a quarter to a half of the general population worry to some extent about getting some type of cancer, and 5% to 10% experience extreme worry.^38^ Altogether, these findings may partially explain the high levels of overdiagnosis women were willing to accept and also underline the importance of providing women with balanced information concerning the benefits and harms of breast cancer screening.

#### 4.2.2 False‐positive findings

European studies show that false‐positive screening results were associated with long‐term negative psychosocial consequences,^20^, ^21^, ^22^ whereas a US study showed only a transient increase in anxiety.^25^ These conflicting results may be related to the different instruments used to measure anxiety. European studies used a screening‐specific validated questionnaire “Consequences of Breast Cancer Screening” specifically developed to assess the long term psychosocial consequences of false‐positive mammography screening, while the US study used the 6 question short‐form (STAI‐6) of the Spielberger State‐Trait Anxiety Inventory (STAI) instrument focusing on measurement of general anxiety. Previous studies have shown inconsistent results concerning psychosocial consequences of false‐positive results, with some women showing persistent and others only transient anxiety.^39^ A systematic review focusing on the UK population reported that receiving a false‐positive screening mammogram caused breast cancer‐specific psychological distress that may endure up to 3 years, and the degree of distress appears to be related to the level of invasiveness of the assessment procedure.^34^ False‐positive results may have substantial other impacts on women's health behaviour and well‐being. Women with false‐positive findings have been shown to make a greater use of health care services and have reported lower quality of life than those without false‐positive findings.^40^

Healthy women at screening age were prepared to accept a high risk of false‐positive screening results in order to detect breast cancer early. Irrespective of false‐positive findings, the screening intention remained high and was even higher among those with a false‐positive result compared with those with a negative result. Despite significant psychosocial burden caused by false‐positive screening results, women acknowledge the value of mammography screening. Our results are consistent with another recent systematic review and meta‐synthesis by the Health Care Ontario, assessing the burden of false‐positive and false‐negative results and their impact on women's screening intentions.^41^

### 4.3 Screening decision among elderly women

Elderly women's preferences regarding breast cancer screening were more heterogeneous. This is consistent with the decreased benefit to risk ratio that these women face.^42^ For these reasons, screening of elderly women is not recommended by the majority of available guidelines.^42^

#### 4.3.1 Diagnostic procedures

The importance of the quick receipt of diagnostic results has been previously emphasised in several studies.^43^, ^44^ A very high number of women would choose to undergo the diagnostic procedure again in the future in order to have the results earlier.^31^ A substantial proportion of women are also willing to accept the inconvenience and anxiety associated with a higher recall rate if it results in earlier breast cancer detection.^23^ Altogether, these findings show that women appear to value more the possibility of an earlier and accurate detection of cancer over the inconvenience and anxiety associated with the diagnostic process itself.

Our results are in agreement with Pahade and coworkers,^45^ who have shown that most patients showed decreased anxiety after receiving the examination results from the radiologist. Although it is generally assumed in clinical practice that the best way for patients to receive diagnostic results is to personally discuss them with a qualified professional, Brandon and colleagues reported that most women (90%) found it acceptable to receive the results even over the phone.^29^ This finding can also partially explain the higher value women place on fast delivery of test results over the method chosen to communicate them.

### 4.4 Study limitations

Our review has several strengths. To the best of our knowledge, this is the first systematic review focusing specifically on women's values and preferences about breast cancer screening and diagnostic services. In our evaluation, we applied rigorous methods including the GRADE approach and CERQual methodology for evidence synthesis and quality evaluation of qualitative results.

The main limitation of our findings relates to the methodological limitations of the included studies. More specifically, we are not confident that the participants of several of the included studies received balanced information in order to understand complex concepts, such as overdiagnosis. Our study is also limited by the relatively small number of studies and small sample size in some of them. Another limitation is that we only included studies published in English. However, the included studies evaluated a wide variety of populations and countries, so we do not believe this limits the generalisation of our findings. The restriction of the search to the last 10 years for primary studies may have also limited our findings, but we are confident that the most important outcomes, such as overdiagnosis, have been mostly studied within this period. The inclusion of previous systematic reviews also limits these concerns. In addition, more recent studies are likely to be more relevant because diagnostic and therapeutic options and outcomes of breast cancer have significantly changed over the last decade.

### 4.5 Clinical implications

The low‐to‐moderate quality of the evidence for breast cancer screening and moderate quality evidence for breast cancer diagnosis underlines the need to carry out more well‐designed studies on women's values and preferences, including also minorities, women with disabilities, with different cultural, religious, educational, and economic backgrounds. Such studies would provide valuable data to panels developing clinical or public health recommendations, as well as to policy‐makers when making coverage or public health decisions.

Health care community should focus on providing clear, adequate, and balanced information on the benefits and risks of breast cancer screening to ensure informed participation. In this context, the use of decision aids could be helpful.^46^ A particular emphasis should be made on the communication of overdiagnosis, which was poorly understood based on our findings. Clinicians should also be encouraged to improve their communication skills and health care systems to provide adequate and timely information about test results.

## ETHICAL BACKGROUND

The European Commission Initiative on Breast Cancer (ECIBC) (http://ecibc.jrc.ec.europa.eu/) uses the GRADE approach when formulating recommendations for breast cancer screening and diagnosis. This includes the use of Evidence to Decision (EtD) frameworks that provide an explicit and transparent system for decision making to ensure all important criteria needed to make a decision are considered. One of these criteria is how those affected by a recommendation value the main desirable and undesirable outcomes of the interventions considered. This systematic review was thus conducted to inform ECIBC's clinical recommendations' development process.

Neither patient consent nor ethical committee approval was necessary as, because of the type of work presented (a systematic review of the literature), this is not needed.

## CONFLICT OF INTERESTS

AM, HPH, IS, and PAC were working at the time when this work was carried out for Iberoamerican Cochrane Collaboration, which received payments from the European Commission to develop the systematic reviews informing the ECIBC recommendations. LP, ZSP, AB, SD, DL, and LN were working, or are working, for the European Commission, JRC. No other interests are declared.

## FUNDING INFORMATION

The systematic review was carried out by Iberoamerican Cochrane Collaboration under Framework contract 443094 for procurement of services between European Commission Joint Research Centre and Asociación Colaboración Cochrane Iberoamericana. Administrative Arrangement SANCO/2012/C – 17.030600/12//SI2.635313 between the European Commission Directorate‐General Health and Food Safety and the Directorate‐General Joint Research Centre. AGM was funded by a Fellowship in Guidelines Methodology by the European Respiratory Society (MTF 2015‐01).

## AUTHORS CONTRIBUTIONS

Alexander Mathioudakis, Ivan Solà, and Pablo Alonso‐Coello were responsible for conducting the systematic review, including the interpretation of the results and the drafting of the full report of the systematic review (available upon request). Alexander Mathioudakis and Hector Pardo‐Hernandez conducted the search and data extraction. Liisa Pylkkanen, Silvia Deandrea, and Donata Lerda contributed to the definition of the research protocol and provided comments to the preliminary results. Alexander Mathioudakis, Pablo Alonso‐Coello, Minna Salakari, and Liisa Pylkkänen drafted the first version of the article. All authors contributed to the interpretation and reporting of the results and provided comments on subsequent versions of the article. All authors read and approved the final manuscript prior submission.

## Supporting information

Data S1. Supporting information

Data S2. Supporting information
