# Supplementary material for: Systematic review on women's values and preferences concerning breast cancer screening and diagnostic services
Source: Psychooncology. 2019 Mar 24;28(5):939–47. doi: 10.1002/pon.5041 (PMC6594004; doi:10.1002/pon.5041)
Supplement: Supplementary file 1 — Data S1. Supporting information [file PON-28-939-s001.docx]

**Appendix 1. Search algorithm and references retrieved**

| **Database and date** | |
| --- | --- |
| **MEDLINE**  Ovid MEDLINE(R) In-Process & Other Non-Indexed Citations, Ovid MEDLINE(R) Daily and Ovid MEDLINE(R) <1946 to Present>  09.12.2015 | 1 *Breast Neoplasms/di (17240)  2 *Breast Neoplasms/ra (6704)  3 breast.ti. (212016)  4 1 or 2 or 3 (218134)  5 *Mass Screening/ (46220)  6 Early Detection of Cancer/ (11933)  7 screen*.ti,ab. (534189)  8 5 or 6 or 7 (546404)  9 4 and 8 (17898)  10 *Mammography/ (15268)  11 mammogra*.ti,ab. (26732)  12 9 or 10 or 11 (38342)  13 diagnos*.ti,ab. (1857062)  14 overdiagnos*.ti,ab. (2622)  15 over diagnos*.ti,ab. (885)  16 overdetection.ti,ab. (75)  17 over detection.ti,ab. (67)  18 13 or 14 or 15 or 16 or 17 (1858213)  19 3 and 18 (37972)  20 12 or 19 (66766)  21 *Choice Behavior/ (11911)  22 *Decision Making/ (31408)  23 *Attitude to Health/ (38689)  24 understanding.ti,ab. (554994)  25 perception*.ti,ab. (171098)  26 preference*.ti,ab. (106827)  27 attitude*.ti,ab. (108337)  28 expectation*.ti,ab. (62344)  29 (value or values).ti,ab. (1410329)  30 (view or views).ti,ab. (280091)  31 informed choice*.ti,ab. (1813)  32 informed decision*.ti,ab. (4662)  33 (women* adj5 decision*).ti,ab. (3170)  34 (screening adj5 decision*).ti,ab. (1777)  35 21 or 22 or 23 or 24 or 25 or 26 or 27 or 28 or 29 or 30 or 31 or 32 or 33 or 34 (2569146)  36 20 and 35 (13444)  37 Qualitative Research/ (25350)  38 Focus Groups/ (19437)  39 qualitative.ti,ab. (142495)  40 interview*.ab. (231234)  41 focus group*.ti,ab. (26987)  42 purposive.ab. (4779)  43 theory.ab. (206388)  44 grounded theory.ab. (7063)  45 (mixed adj3 method*).ti,ab. (9327)  46 meta-ethnograph*.ti,ab. (227)  47 meta-synthe*.ti,ab. (337)  48 37 or 38 or 39 or 40 or 41 or 42 or 43 or 44 or 45 or 46 or 47 (547218)  49 36 and 48 (1316)  50 limit 49 to "systematic reviews" (28)  51 limit 49 to "reviews (best balance of sensitivity and specificity)" (75)  52 50 or 51 (85)  53 49 not 52 (1231)  54 36 not 49 (12128)  55 exp Decision Support Techniques/ (66253)  56 (health adj3 utilit*).ti,ab. (2466)  57 gamble*.ti,ab. (3318)  58 prospect theor*.ti,ab. (174)  59 preference score*.ti,ab. (373)  60 (preference* adj5 elicitat*).ti,ab. (165)  61 health utilit*.ti,ab. (1362)  62 (utilit* adj3 (value* or score* or estimate*)).ti,ab. (3224)  63 (state adj5 utilit*).ti,ab. (773)  64 health state.ti,ab. (2958)  65 feeling thermometer*.ti,ab. (54)  66 best-worst scaling.ti,ab. (63)  67 standard gamble.ti,ab. (740)  68 time trade-off.ti,ab. (889)  69 TTO.ti,ab. (721)  70 probability trade-off.ti,ab. (16)  71 55 or 56 or 57 or 58 or 59 or 60 or 61 or 62 or 63 or 64 or 65 or 66 or 67 or 68 or 69 or 70 (77125)  72 54 and 71 (154)  73 54 not 72 (11974)  74 *Questionnaires/ (33072)  75 Cross-Sectional Studies/ (209337)  76 (survey or questionnair* or cross-sectional).ti,ab. (784271)  77 74 or 75 or 76 (858049)  78 73 and 77 (1486)  79 limit 78 to "systematic reviews" (29)  80 limit 78 to "reviews (best balance of sensitivity and specificity)" (50)  81 79 or 80 (64)  82 78 not 81 (1422)  83 73 not 82 (10552)  84 review.pt. (2087807)  85 83 and 84 (1226)  86 52 or 53 or 72 or 81 or 82 or 85 (4144) |
