# Supplementary material for: Systematic review on women's values and preferences concerning breast cancer screening and diagnostic services
Source: Psychooncology. 2019 Mar 24;28(5):939–47. doi: 10.1002/pon.5041 (PMC6594004; doi:10.1002/pon.5041)
Supplement: Supplementary file 2 — Data S2. Supporting information [file PON-28-939-s002.docx]

**Appendix 2. Evidence Profiles.**

***Screening decisions***

| **Quality assessment** | | | | | | | **Study population and characteristics** | **Findings** | **Quality** | **Studies Contributing to the Review Finding** |
| --- | --- | --- | --- | --- | --- | --- | --- | --- | --- | --- |
| **No. of studies** | **Study design** | **Risk of bias/Methodological limitations** | **Inconsistency/Coherence** | **Indirectness/Relevance** | **Imprecision/Adequacy of data** | **Other considerations** |  |  |  |  |
| ***Overdiagnosis*** | | | | | | | | | | |
| 3 | Cross-sectional studies | serious^1^ | none | non serious^2^ | none | none | 3216 adult women | The disutility of overdiagnos was high among women in the included studies. However, they consider it an acceptable risk of mammographic breast cancer screening. | ⨁⨁◯◯ LOW | Baena-Cañada 2014, Van den Bruel 2015, Waller 2014 |
| 2 | Qualitative studies | serious^1^ | none | none | serious^3^ | none | 90 women of breast screening age |  | ⨁⨁◯◯ LOW | Hersch 2013, Waller 2013 |
| ***False positives*** | | | | | | | | | | |
| 4 | Cross-sectional studies | non serious^3^ | none | none | none | none | 1173 women with a false positive mammographic result versus 1424 women with a negative mammographic result. | The disutility of false positive mammographic results was high among women in the included studies. However they consider it an acceptable risk of mammographic breast cancer screening. | ⨁⨁⨁◯ MODERATE | Bolejko 2015, Brodersen 2013, Ganott 2006, Tosteson 2014, Vass 2018 |
| 2 | Qualitative studies | serious^1^ | none | Serious^5^ | none | none | 53 women who have received a false positive mammographic result. |  | ⨁⨁◯◯ LOW | Bolejko 2014, Thompson 2013 |
| ***Burden associated with breast cancer screening programmes*** | | | | | | | | | | |
| 1 | Systematic review of 21 qualitative studies | non serious^5^ | none | non serious | non serious^6^ | none | 1084 women of different ethnicities. USA (8), Iran (3), Jordan (1), Black minority ethnic groups in the UK, Greece (1), Western Cape (1), Malaysia (1), Turkey (1), Spain (1), Chile (1), United Arab Emirates (1). | Logistical implications of breast cancer screening, including time and money expenditure, may be burdensome for women who participate in these screening programs. | ⨁⨁⨁◯ MODERATE | Azami-Aghdash 2015 |

^1^ Concerns regarding adequacy of information given to participants in order to take an informed decision.

^2^ Questionnaires were distributed among adult women in general, rather than women of screening age.

^3^ Only limited data are available.

^4^ Values of patients who have already received a false positive result were assessed.

^5^ The included studies were subject to minor methodological limitations. Authors do not link findings and the studies they were derived from.

^6^ Only three of the included studies were conducted in Europe. Authors also acknowledge that some of the review findings were highlighted in <25% of the included studies. ***Diagnostic decisions***

| **Quality assessment** | | | | | | | | **Study population and characteristics** | **Findings** | **Quality** | **Studies Contributing to the Review Finding** |
| --- | --- | --- | --- | --- | --- | --- | --- | --- | --- | --- | --- |
| **No. of studies** | **Study design** | **Risk of bias/Methodological limitations** | | **Inconsistency/Coherence** | **Indirectness/Relevance** | **Imprecision/Adequacy of data** | **Other considerations** |  |  |  |  |
| ***Anxiety*** | | | | | | | | | | | |
| 1  4 | SR of cross sectional studies  Cross sectional studies | | None | none | none | serious^1^ | none | 3056 breast cancer patients. | Patients highly disvalue avoidable anxiety. | ⨁⨁⨁◯ MODERATE | Fiszer 2014, Brandon 2011, Chicken 2007, Miller 2013, Presutti 2014 |
| ***Inconvenience*** | | | | | | | | | | | |
| 1 | Cross sectional study | None | | none | none | serious^1^ | none | 49 women who underwent both CESM and CEMRI as part of a trial in Australia. | Women highly value comfortable and fast procedure. | ⨁⨁⨁◯ MODERATE | Hobbs 2015 |

^1^ Inadequate data.
